# Medicinal polypharmacology—a scientific glossary of terminology and concepts

**DOI:** 10.3389/fphar.2024.1419110

**Published:** 2024-07-18

**Authors:** Sven Marcel Stefan, Muhammad Rafehi

**Affiliations:** ^1^ Medicinal Chemistry and Systems Polypharmacology, Medical Systems Biology Division, Lübeck Institute of Experimental Dermatology (LIED), University of Lübeck and University Medical Center Schleswig-Holstein (UKSH), Lübeck, Germany; ^2^ Department of Biopharmacy, Medical University of Lublin, Lublin, Poland; ^3^ Institute of Clinical Pharmacology, University Medical Center Göttingen, Göttingen, Germany; ^4^ Department of Medical Education, Augsburg University Medicine, Augsburg, Germany

**Keywords:** polypharmacolome, drug repurposing, target repurposing, privileged structures, privileged ligands, network pharmacology, chemogenomic space, superfolds

## Abstract

Medicinal polypharmacology is one answer to the complex reality of multifactorial human diseases that are often unresponsive to single-targeted treatment. It is an admittance that intrinsic feedback mechanisms, crosstalk, and disease networks necessitate drugs with broad modes-of-action and multitarget affinities. Medicinal polypharmacology grew to be an independent research field within the last two decades and stretches from basic drug development to clinical research. It has developed its own terminology embedded in general terms of pharmaceutical drug discovery and development at the intersection of medicinal chemistry, chemical biology, and clinical pharmacology. A clear and precise language of critical terms and a thorough understanding of underlying concepts is imperative; however, no comprehensive work exists to this date that could support researchers in this and adjacent research fields. In order to explore novel options, establish interdisciplinary collaborations, and generate high-quality research outputs, the present work provides a first-in-field glossary to clarify the numerous terms that have originated from various individual disciplines.

## 1 Medicinal polypharmacology—an introduction

Drug development has evolved over the last decades and is increasingly acknowledging integral approaches to achieve clinical effectiveness of drugs, specifically taking target combinations into account (Morphy and Rankovic, 2007; Zimmermann et al., 2007; Proschak et al., 2019). Although the majority of academic research and industrial efforts still adhere to the Specificity paradigm (One drug-one target philosophy) (Morphy and Rankovic, 2007; Jalencas and Mestres, 2013a; Anighoro et al., 2014), the general perception of human pathology is changing to a more integral point of view acknowledging the existence of complex coherences in malignant, metabolic, or neurological diseases that may be engaged simultaneously (Morphy and Rankovic, 2007; Anighoro et al., 2014; Proschak et al., 2019) to overcome ineffective pharmacotherapy (Anighoro et al., 2014; Proschak et al., 2019).

This vastly growing research field, which we designated as “medicinal polypharmacology” (Stefan and Rafehi, 2023; Rafehi et al., 2024), expands into various individual research fields, allowing for novel concepts to emerge.

(i) The clinically observed effects of drugs are generally a result of multiple individual interactions with multiple interaction partners (Paolini et al., 2006; Vulpetti et al., 2012; Jalencas and Mestres, 2013b; Anighoro et al., 2014; Schmidt et al., 2014);
(ii) Prevalent human diseases are often multifactorial with far-reaching disease networks that result in feedback, crosstalk, and, subsequently, therapy resistance (Morphy and Rankovic, 2007; Azmi and Mohammad, 2014; Keith et al., 2005);
(iii) Multitargeticity is an inherent character of small molecules that has molecular-structural limits (Paolini et al., 2006; Hu and Bajorath, 2010; Jalencas and Mestres, 2013a; Anighoro et al., 2014; Namasivayam et al., 2022a);
(iv) Phylogenetically distant proteins have common and reoccurring structural motifs [Superfolds (Orengo et al., 1994; Russell et al., 1998; Grishin, 2001; Koch, 2011)] which can form Supersites that bind related, multitarget ligands (Russell et al., 1998; Namasivayam et al., 2021a);
   (v)  The multitarget inheritance of multitarget drugs allows for superior clinical effectiveness and, in parallel, exploration of yet undruggable targets of the future [Privileged ligands (Jalencas and Mestres, 2013a; Kim et al., 2014) and Target repurposing (Paolini et al., 2006; Pollastri and Campbell, 2011; Klug et al., 2016; Singh et al., 2020)].

## 2 The challenge of a common language—filling an important gap in medicinal polypharmacology with a first-in-field glossary

Medicinal polypharmacology is an attractive multidisciplinary research field that bears a certain linguistic complexity. Multitarget paradigm, Supersites, Privileged ligands, and Target repurposing—these terms are nothing but words unless an interconnected explanation is provided. In order to discuss medicinal polypharmacology and the individual aspects involved, an understanding of the terminology and developed concepts is necessary. This is particularly true with respect to how these terms and concepts are embedded in the broader context of medicinal chemistry, chemical biology, and clinical pharmacology.

Instances in the past have demonstrated that incorrect terminologies can lead to misleading interpretations in medical life sciences. These include, for example, the use of incorrect terms and units (Zavorsky, 2021), incorrect physiological and anatomical descriptions (Franz-Odendaal, 2023), or erroneous histopathological interpretations that can lead to false diagnoses (Brunye et al., 2023). These and other instances have resulted in article corrections (Judge et al., 2020), commentaries and letters to the editor (Zavorsky, 2021), entire review articles addressing specific incorrect terminology (Tao et al., 2023), and subject-specific online glossaries. Although it is undoubtedly important to clarify misconceptions, it would even be better to avoid them altogether. Clear and precise language is important for communication, discussion, and awareness, which prompted us to provide a first-in-field glossary of terms and concepts used in medicinal polypharmacology. This glossary takes the pioneering and contemporary literature, the standardized nomenclature as outlined by IUPAC (Proudfoot et al., 2011) and IUPHAR, as well as definitions from medical subject headings (MeSH; https://www.ncbi.nlm.nih.gov/mesh) into account:

### Antitarget

An Off-target effect that leads to severe adverse events and/or toxicity (McKie, 2016; Ravikumar and Aittokallio, 2018; Polanski and Bak, 2019).

### Applicability domain

Chemical and biological space toward which (computer-driven) predictions are feasible and reliable based on a training set of ligands/small-molecule drugs; in polypharmacology, the multiplicity of data (origin) and controversial/contradicting data (*e.g.*, Promiscuity cliffs) as well as publication bias and data pollution hamper the prediction capabilities and limit the applicability domain of computational prediction methodologies (Taula et al., 2009; Stefan and Rafehi, 2024).

### Assay validation

Experimental verification that the measured output (readout) of an assay is in association with, and correlates to, the biological activity of the anticipated target (consistent reflection); assay validation is a valuable part of a Target validation and subsequent Lead identification processes at the very beginning of the drug development pipeline (Proudfoot et al., 2011).

### Bad actor

Compound that interferes with assay readouts and causes artifacts and, thus, leads to its identification as a false-positive hit (Stork and Kirchmair, 2018).

### Balancing

Modulating the biological activity of a polypharmacological ligand/small-molecule drug against two or more targets to achieve an optimal ratio of activities, and thus, a preferable polypharmacological profile; optimized polypharmacological ligands/small-molecule drugs are also referred to as “balanced”; balancing is achieved by Designing-in drug design and Designing-out drug design (Morphy et al., 2004; Morphy and Rankovic, 2006; Korcsmaros et al., 2007; Morphy and Rankovic, 2007; Morphy and Rankovic, 2009; Zhan and Liu, 2009; Peters, 2013; Proschak et al., 2019).

### Bioactivity space

*see* Space.

### Cellular networks

Networks of genes, the cytoskeleton, the metabolism, organelles, proteins, and signaling connected by bridges, hubs, links, and critical nodes offering potential druggable binding sites; also referred to as Interactome; cellular networks are studied in the discipline of Network biology (Barabasi and Oltvai, 2004; Hopkins et al., 2006; Korcsmaros et al., 2007; Morphy and Rankovic, 2009; McKie, 2016).

### Chemical promiscuity

*see* Promiscuity.

### Chemical space

*see* Space.

### Chemical tool

Ligand/small-molecule drug designed or developed to modulate and analyze the function of the target(s) of interest; chemical tools are important factors in the Target validation process at the very beginning of the drug development pipeline (Hopkins et al., 2006).

### Cheminformatics

Computer-aided data mining and acquisition to comprehend and address current problems in chemistry; cheminformatics is strongly related to Chemometrics.

### Chemogenomic space

*see* Space.

### Chemoisosterism

The property of structurally, functionally, and/or phylogenetically distant proteins to interact with the same chemical fragments/entities/scaffolds; related to Supersites (Russell et al., 1998; Jalencas and Mestres, 2013b; Anighoro et al., 2014).

### Chemometrics

Chemical discipline to extract and/or maximize chemical information and knowledge in chemical systems by (computer-driven) mathematical and statistical methodologies; chemometrics is strongly related to Cheminformatics and overlaps with the discipline of Systems chemistry; chemometrics is used, for example, for Quantitative structure-activity relationships (QSARs) (Proudfoot et al., 2011; Joshi, 2023).

### Chemotypes

Chemical class/structure/scaffold; Virtual screening and High-throughput screening (HTS) anticipate the discovery of molecular-structurally and/or functionally novel chemotypes, particularly in medicinal polypharmacology; chemotypes are also referred to as “topologically equivalent scaffolds” that result in the same carbon skeleton (Hopkins et al., 2006; Zhan and Liu, 2009; Anighoro et al., 2014; Proschak et al., 2019).

### Combinatorial chemistry

Organic synthesis approach to generate a virtually unlimited number of novel, drug-like molecules out of a limited set of starting materials.

### Combination drug therapy (CTD)

*see* Polypharmacy (Kim et al., 2014).

### Compound-based target relationships (CBTRs)

Two or more targets share a defined number of ligands/small-molecule drugs; these Privileged ligands provide the basis for novel drug Target identification and Target validation; CBTRs are the structure-based counterpart of Structure–activity relationships (SARs) (Bajorath, 2021).

### Computer-aided pattern analysis (C@PA)

A computational fragment-based methodology for 1) the extraction of Multitarget fragments and, subsequently, Multitarget fingerprints from a set of compounds that were functionally evaluated against two or more targets, and 2) the application of these Multitarget fragments and Multitarget fingerprints against chemical space to predict polypharmacological ligands; also referred to as Computer-aided pattern scoring (C@PS) (Namasivayam et al., 2021a; Namasivayam et al., 2021b; Namasivayam et al., 2022b; Stefan et al., 2022; Stefan et al., 2023; Stefan et al., 2024).

### Computer-aided pattern scoring (C@PS)

*see* Computer-aided pattern analysis (C@PA).

### Constellation pharmacology

The approach to uncover Target constellations (Wang and Yang, 2022).

### Cross-pharmacology

Ability of two or more phylogenetically distant targets to share the same set of ligands/small-molecule drugs; also referred to as Distant polypharmacology (Jalencas and Mestres, 2013a; Jalencas and Mestres, 2013b; Anighoro et al., 2014; Antolin and Mestres, 2015).

### Cross-reactivity

*see* Unselectivity.

### Cross-screening

*see* High-throughput screening (HTS).

### Dark chemical matter

A group of compounds that has extensively been evaluated without notable biological activity and which may lead to hit molecules toward yet unknown or barely established novel drug targets by exceptional, unexpected, and/or unprecedented bioactivity, modes-of-action, and Specificity (Wassermann et al., 2015).

### Deorphanization

An approach to explore Orphan targets (Franchini and Orlandi, 2023).

### Design-in approach

*see* Designing-in drug design.

### Design-out approach

*see* Designing-out drug design.

### Designed multiple ligand (DML)

Ligand/small-molecule drug designed to address multiple targets (Morphy and Rankovic, 2005; Morphy and Rankovic, 2009).

### Designing-in drug design

Knowledge-based (Framework combination) design of a novel molecular-structural entity out of two or more selective (and potent) ligands/small-molecule drugs against one or more independent targets to obtain simultaneous biological activity against all targets of interest within one molecule; also referred to as Design-in approach (Morphy et al., 2004; Zhan and Liu, 2009; Proschak et al., 2019).

### Designing-out drug design

Design of a novel molecular-structural entity without undesired biological activity from a template molecule that has both the desired biological activity against one or more targets as well as undesired biological effects; also referred to as Design-out approach (Morphy and Rankovic, 2009; Zhan and Liu, 2009; Proschak et al., 2019).

### Difficult-to-drug target

*see* Orphan target.

### Dirty drug

A drug that exerts multiple Off-target effect, potentially leading to adverse events; like Promiscuity, the term Dirty drug has a negative connotation (Azmi and Mohammad, 2014).

### Diseasome

Disease-related Interactome (Wang and Yang, 2022).

### Distant polypharmacology

The polypharmacological nature of a ligand/small-molecule drug against two or more phylogenetically distant targets (Jalencas and Mestres, 2013b; Antolin and Mestres, 2015).

### Druggability

The accessibility of a target by ligands/small-molecule drugs (Vulpetti et al., 2012).

### Drug annotation

*see* Drug profiling.

### Drug cocktail

*see* Polypharmacy.

### Drug likeness

Preferable molecular-structural and physicochemical properties originally defined by Lipinski et al. (2001); also referred to as Lipinski-rule-of-five. According to the authors’ original definition, developed drugs should anticipate a calculated octanol-water partition coefficient (CLogP) ≤ 5, a molecular weight (MW) ≤ 500 g mol^−1^, not more than five hydrogen-(H)-bond donors, and not more than 10 H-bond acceptors; molecular-structural and physicochemical properties are particularly important in the design of polypharmacological ligands/small-molecule drugs (Lipinski et al., 2001; Namasivayam et al., 2022a).

### Drug profiling

Annotation of a drug (Drug annotation) to physical, chemical, and/or biological attributes; in the drug development pipeline, drug profiling is used to uncover unknown and/or unwanted Off-target effects; many automated design tools exist to predict and map polypharmacological profiles (Besnard et al., 2012; Bowes et al., 2012; Schmidt et al., 2014; Ravikumar and Aittokallio, 2018; Popovic et al., 2019).

### Drug redirecting

*see* Drug repurposing.

### Drug rediscovering

*see* Drug repurposing.

### Drug repositioning

*see* Drug repurposing.

### Drug reprofiling

*see* Drug repurposing.

### Drug repurposing

Deliberate and methodical exploration of novel therapeutic purposes and/or indications for existing/approved drugs; also referred to as Drug repositioning, Drug reprofiling, Drug redirecting, Drug retasking, Drug rediscovering, or Rescue of drugs (Peters, 2013; Anighoro et al., 2014; Azmi and Mohammad, 2014; McKie, 2016; Santos et al., 2016; Bajorath, 2022; Wang and Yang, 2022).

### Drug retasking

*see* Drug repurposing.

### First-in-class drug

Drug that constitutes the first member of a chemical or pharmacological class of bioactive agents (Kim et al., 2014).

### Framework combination

Knowledge-based approach for designing polypharmacological ligands/small-molecule drugs based on the combination of molecular-structural frameworks and pharmacophores of selective (and potent) molecules toward the targets of interest; also referred to as Designing-in drug design or Design-in approach (Morphy et al., 2004; Morphy and Rankovic, 2006; Morphy and Rankovic, 2007; Morphy and Rankovic, 2009; Zhan and Liu, 2009; Proschak et al., 2019).

### Frequent hitter

Compound with a higher-than-expected hit rate in various assays (Hopkins et al., 2006; Jalencas and Mestres, 2013a; Stork and Kirchmair, 2018; Wang and Yang, 2022).

### Functional promiscuity

*see* Promiscuity.

### High-content screenings

*see* Phenotypic screening.

### High-throughput screening (HTS)

Large-scale (random) screening of preferably multiple (and diverse) analogous/physical compound libraries in a short time frame; in Polypharmacology, these screenings are carried out against multiple targets (Cross-screening) in order to identify molecular-structurally (and/or functionally) novel hit molecules (Chemotypes) with polypharmacological profiles; HTS is based on Serendipity (Morphy et al., 2004; Morphy and Rankovic, 2006; Morphy and Rankovic, 2009; Zhan and Liu, 2009; Proudfoot et al., 2011; Ravikumar and Aittokallio, 2018).

### Interactome

The physical molecular interactions between ligands and targets, including associated proteins and/or genes, as well as biochemical cascades, crosstalk, and feedback; also referred to as Cellular network; the interactome is studied in the discipline of Network biology (Barabasi and Oltvai, 2004; Hopkins et al., 2006; Korcsmaros et al., 2007; Morphy and Rankovic, 2009; McKie, 2016).

### Landscape

Defined and charted inventory of ligands/small-molecule drugs, partial structures and functional groups, targets, and/or bioactivities (Anighoro et al., 2014; Namasivayam et al., 2022b; Stefan et al., 2022; Wu et al., 2022; Puri et al., 2023).

### Lead identification

Identification of a ligand for (a) particular target(s) of interest that fulfills the requirements for the subsequent drug development stage of Lead optimization; lead molecules are usually discovered via High-throughput screening (HTS) (Proudfoot et al., 2011).

### Lead optimization

Improvement of the biological activities and other relevant properties (*e.g.*, safety profile, drug likeness, *etc.*) of a lead molecule with medicinal chemistry methodologies; in Polypharmacology, lead optimization is achieved by Balancing, Designing-in drug design, and Designing-out drug design (Proudfoot et al., 2011):

### Lead repurposing

Exploration of the extended pharmacological profile of a lead compound of the early drug development stage initially designed/discovered to target one particular target (class) only (Klug et al., 2016; Singh et al., 2020).

### Ligand fishing

Experimental setup using immobilized targets of interest to identify novel binding partners by, for example, mass spectrometry, quantitative proteomics, or microarrays; ligand fishing is an integral part of the drug development pipeline and can be seen as reciprocal to Target fishing, *in vitro* (Chen et al., 2020).

### Ligand-based drug design

Computer-aided extraction of information about descriptors and fingerprints present in a set of ligands/small-molecule drugs with demonstrated interaction with, or biological activity against, the target(s) of interest; ligand-based drug design is supported by Cheminformatics (Proudfoot et al., 2011; Proschak et al., 2019).

### Lipinski-rule-of-five

*see* Drug likeness.

### Magic bullet

Obsolete synonym for a highly potent, specific, and selective ligand/small-molecule drug that interacts with one defined target only; the opposite of Magic shotgun (Morphy et al., 2004; Terstappen et al., 2007; Anighoro et al., 2014; Bansal and Silakari, 2014; Ravikumar and Aittokallio, 2018).

### Magic shotgun

Obsolete synonym for a polypharmacological ligand/small-molecule drug; the opposite of Magic bullet (Ravikumar and Aittokallio, 2018).

### Matched molecular pair

*see* Promiscuity cliffs.

### Morphological profiling

Target-independent screening methodology based on cell painting assays using various dyes to image the morphological size, shape, and texture of cellular and organellar components with high-throughput microscopy under exposure of ligands/small-molecule drug candidates; morphological profiling provides spectral fingerprints that may be compared to reference compounds for the identification of similar (or distinctive) targets and/or modes-of-action (Bray et al., 2016; Schneidewind et al., 2020).

### Multicomponent drug

*see* Polypharmacy.

### Multifactorial compounds

Hybridization of individual ligands/small-molecule drugs to a hybrid drug (“conjugated”) or chimeric drug (“fused”) (Bansal and Silakari, 2014).

### Multimeric ligands

Multiple monomeric ligands (individual ligands/small-molecule drugs) attached to a single backbone (small-)molecule; very close to the definition of a conjugate (*see* Polypharmacology) (Handl et al., 2004).

### Multitargeticity

The ability of a ligand/small-molecule drug to address more than one target; also referred to as Multitargeting; it is commonly accepted that multitargeticity is given when targets of different, phylogenetically distant target (super)families are addressed (Stefan, 2019).

### Multitargeting

*see* Multitargeticity.

### Multitarget fingerprints

A sum of measured properties and/or mathematically calculated descriptors that conserve chemical, molecular-structural, and/or physicochemical attributes of molecules/partial structures/functional groups that are associated with biological effects toward multiple targets; ultimately distinguished from single-target descriptors/fingerprints (Polanski and Bak, 2019).

### Multitarget drug (MTD) concept

*see* Multitarget paradigm.

### Multitarget fragments

Molecular-structural attribute of partial structures/functional groups that are associated with biological effects toward multiple targets (Brunst et al., 2021).

### Multitarget paradigm

The concept that a ligand/small-molecule drug interacts with multiple targets to exert its biological effect(s); also referred to as the Multitarget drug (MTD) concept (Medina-Franco et al., 2013; Santos et al., 2016; Ravikumar and Aittokallio, 2018; Wang and Yang, 2022).

### Network biology

Discipline addressing the biological organization of cellular components, such as protein–protein interactions, metabolic, signaling, and transcription regulation, as well as Cellular networks; also covered by the term Interactome (Barabasi and Oltvai, 2004; Hopkins et al., 2006; Morphy and Rankovic, 2009; McKie, 2016).

### Network pharmacology

Pharmacological intervention with a small-molecule drug under consideration and understanding of the relevant Cellular network and Interactome; network pharmacology also anticipates the prediction, discovery, and identification of optimal target combinations; also referred to as Systems pharmacology (Hopkins, 2008; Proschak et al., 2019; Wang and Yang, 2022).

### Non-selectivity

*see* Unselectivity.

### Non-specific effect

*see* Unspecific effect.

### Nuisance compound

Low-quality compound that interferes with assay readouts, consumes precious scientific resources, and leads to publication bias (Stork and Kirchmair, 2018; Dahlin et al., 2021).

### Off-target effects

Unintended effect of a ligand/small-molecule drug on a target irrelevant to the respective disease; off-target effects are believed to be a major reason for (secondary) adverse events (also called Side effects) and toxicity (Hopkins et al., 2006; Morphy and Rankovic, 2006; Morphy and Rankovic, 2007; Bowes et al., 2012; Vulpetti et al., 2012; Peters, 2013; Schmidt et al., 2014; McKie, 2016; Polanski and Bak, 2019; Proschak et al., 2019; Wang and Yang, 2022).

### On-target effect

Intended effect of a ligand/small-molecule drug on an anticipated drug target; polypharmacology anticipates multiple on-targets (Carragher et al., 2012; Wang and Yang, 2022).

### One molecule-one target-one disease philosophy

*see* Specificity paradigm.

### One target-one disease philosophy

*see* Specificity paradigm.

### One target-one drug model

*see* Specificity paradigm.

### Orphan target

Undruggable target for which no ligands/small-molecule drugs are available; also referred to as Difficult-to-drug target or Yet-to-be-drugged target (Wassermann et al., 2009; Coleman and Rodon, 2021; Franchini and Orlandi, 2023).

### PAINS

Pan-assay interference compounds that result in false-positive hits in various assays; PAINS are, to a certain extent, predictable (Ravikumar and Aittokallio, 2018; Stork and Kirchmair, 2018; Wang and Yang, 2022):

### Pan-targets

*see* Redundant pathways.

### Pan-modulation

The modulating [*e.g.*, activating/agonizing, inhibiting/antagonizing, (up-/down-)regulating, *etc*.], nature of a ligand/small-molecule drug toward a panel of related targets belonging to a common target (sub/super)family (Namasivayam et al., 2021c).

### Pharmacophore

Three-dimensional molecular-structural motif of a small molecule necessary to act as a ligand of the target(s) of interest.

### Phenotypic screenings

Screening of potential ligands/small-molecule drugs for particular *in vitro* and/or *in vivo* biological effects with multidimensional readouts; also referred to as High-content screening (Proudfoot et al., 2011; Kim et al., 2014; Proschak et al., 2019).

### Polypharmacodynamics

Analysis of the therapeutic effectiveness of multiple drugs on a biological system (Wang and Yang, 2022).

### Polypharmacokinetics

Analysis of absorption, distribution, metabolism, and excretion of multiple drugs in a biological system (Wang and Yang, 2022).

### Polypharmacology

The interaction of a single (polyvalent) drug with multiple targets and/or disease pathways to treat a pathological condition; polypharmacology is related to the positive connotation of promiscuity; the single drug can be a hybrid/chimeric compound (conjugate) of individual drugs connected by a (potentially cleavable) linker or a genuinely multitarget single agent that is either fused (pharmacophore overlap) or merged (pharmacophore integration); polypharmacological ligands/small-molecule drugs can also be referred to as Multimeric ligands or Multifactorial compounds; the anticipated development of polypharmacological ligands/small-molecule drugs is referred to as Targeted polypharmacology (Handl et al., 2004; Morphy et al., 2004; Morphy and Rankovic, 2005; Hopkins et al., 2006; Morphy and Rankovic, 2006; Korcsmaros et al., 2007; Vulpetti et al., 2012; Jalencas and Mestres, 2013b; Medina-Franco et al., 2013; Peters, 2013; Anighoro et al., 2014; Bansal and Silakari, 2014; Santos et al., 2016; Polanski and Bak, 2019; Proschak et al., 2019; Wang and Yang, 2022).

### Polypharmacolome

The opportunity space of molecular interactions between the structural-biological limitation of target proteins (Structural conservatism of nature) and the molecular-structural limitation of multitarget ligands/small-molecule drugs (Multitarget fingerprint; Superpatterns) (Stefan and Rafehi, 2023; Rafehi et al., 2024).

### Polypharmacy

Combined use of multiple (single-targeted) drugs to treat a pathological condition with combined effects; also referred to as Combination drug therapy (CTD), Drug cocktail (individual drugs), or Multicomponent drug (coformulated drugs); polypharmacy anticipates the use of drugs with different modes-of-action to achieve Selective synergy (increased efficacy at reduced adverse effects); also referred to as Polypharmacotherapy (Morphy and Rankovic, 2005; Korcsmaros et al., 2007; Carragher et al., 2012; Jalencas and Mestres, 2013a; Peters, 2013; McKie, 2016; Wang and Yang, 2022).

### Polypharmacotherapy

*see* Polypharmacy.

### Polyspecificity

*see* Promiscuous target.

### Privileged ligands

Molecular frameworks and entities (Superpatterns) and/or high-quality compound collections that conserve diverse biological activities, which “cross-over” target protein (super)families, and may be used to address yet undruggable targets; often referred to as “rich sources of chemical diversity” with “broad-range biological activity”; furthermore, privileged ligands can be defined as high-quality compound collections of functionally diverse drugs with diverse molecular targets to obtain an optimized Combination drug therapy (CTD) in Phenotypic screenings (High-content screenings) to overcome resistance and/or to acknowledge and address the multifactoricity of diseases (Morphy et al., 2004; Duarte et al., 2007; Zimmermann et al., 2007; Proudfoot et al., 2011; Jalencas and Mestres, 2013a; Kim et al., 2014; Stork and Kirchmair, 2018; Li et al., 2022; Wang and Yang, 2022; Stefan and Rafehi, 2023; Tolomeu and Fraga, 2023).

### Privileged structures

*see* Superpatterns.

### Privileged scaffolds

*see* Superpatterns.

### Promiscuity

A multitarget character of a ligand/small-molecule drug that includes both the therapeutic effects through interaction with the intended targets and the adverse events from Off-target effects; in polypharmacological drug design, it is also referred to as Chemical promiscuity, Functional promiscuity, and Strategic promiscuity; promiscuity is nowadays considered to be a rather negative characteristic (Stark, 2004; Hopkins et al., 2006; Vulpetti et al., 2012; Jalencas and Mestres, 2013a; Peters, 2013; Anighoro et al., 2014; Polanski and Bak, 2019; Bajorath, 2022; Wang and Yang, 2022).

### Promiscuity cliff

Strong difference in activities between molecules of close molecular-structural similarity (Matched molecular pair) toward multiple targets (McKie, 2016).

### Promiscuous target

A target with susceptibility to be addressed by various different compound classes; also referred to as Polyspecificity (Hopkins et al., 2006; Degiacomi et al., 2020; Wang and Yang, 2022).

### Protein structure similarity clustering

Clustering of secondary protein structures around the ligand-sensing cores (secondary protein structure of binding sites and/or catalytic centers) to identify common supra-structural motifs and Supersites (Russell et al., 1998; Koch et al., 2004; Koch, 2011).

### Quantitative structure-activity relationships (QSARs)

Computer-aided prediction of biological activity of small molecules based on Structure–activity relationships (SARs) data.

### Redundant pathways

Pathways and/or signaling cascades that consist of proteins from the same protein family with similar biological outcomes; also referred to as Pan-targets (Hopkins, 2008; Vulpetti et al., 2012; Stefan et al., 2020; Wang and Yang, 2022).

### Repurposome

Network of repurposed drugs, including their annotated targets and bioactivities (Cavalla and Crichton, 2023).

### Rescue of drugs

*see* Drug repurposing.

### Screen validation

Establishment of assay parameters as determined in the Assay validation in a High-content screening format (Proudfoot et al., 2011).

### Selective synergy

*see* Polypharmacy.

### Selectivity

Ability of a ligand/small-molecule drug to exclusively modulate the target of interest compared to other, phylogenetically close or distant (alternative) targets or target classes; it is dependent on Target profiling assays with large numbers of assessed targets; it shall be noted that true (global) selectivity does not exist (Proudfoot et al., 2011; Jalencas and Mestres, 2013b; Antolin and Mestres, 2015; Bajorath, 2021).

### Serendipity

Unintended but fortunate discovery; to date, most discoveries in Polypharmacology were serendipitous, with retrospective elucidation of modes-of-modulation (Morphy et al., 2004; Morphy and Rankovic, 2005; Morphy and Rankovic, 2007; Ravikumar and Aittokallio, 2018; Proschak et al., 2019; Bajorath, 2022; Wang and Yang, 2022).

### Side effects

*see* Off-target effect.

### Single-target drug (STD) concept

*see* Specificity paradigm.

### Space

Includes both the charted and uncharted inventory of ligands/small-molecule drugs, partial structures and functional groups, targets, and/or bioactivities; space refers to what is available and what could (potentially) be available on the chemical (Chemical space) and biological levels (Target space and Bioactivity space); often referred to as Chemogenomic space in which chemical and binding site similarities/conserved target motifs are linked (Koch, 2011; Vulpetti et al., 2012; Fechner et al., 2013; Mousavian and Masoudi-Nejad, 2014; Namasivayam et al., 2022b; Stefan et al., 2022; Wu et al., 2022; Puri et al., 2023).

### Specificity

The degree by which the observed effect in a biological testing system is caused by the specific interaction between a ligand/small-molecule drug and the target (class) of interest.

### Specificity paradigm

The concept that a drug (candidate) should address one target only; also referred to as One target-one drug model, Single-target drug (STD) concept, One target-one disease philosophy, or One molecule-one target-one disease philosophy (Morphy et al., 2004; Morphy and Rankovic, 2005; Hopkins et al., 2006; Zimmermann et al., 2007; Morphy and Rankovic, 2009; Jalencas and Mestres, 2013a; Medina-Franco et al., 2013; Peters, 2013; Santos et al., 2016; Bajorath, 2022; Wang and Yang, 2022).

### Strategic promiscuity

*see* Promiscuity.

### Structural conservatism of nature

The principle that spatial structure is more conserved than amino acid sequences in nature, and that the number of fold types for protein domains is limited (Russell et al., 1998; Grishin, 2001; Koch et al., 2004).

### Structure–activity relationships (SARs)

Correlation between molecular-structural specificities of ligands/small-molecule drugs and their exerted biological effects (Proudfoot et al., 2011).

### Structure-based drug design

Computer-aided extraction of information about descriptors and fingerprints deduced from ligands/small-molecule drugs co-crystallized/bound to the target(s) of interest in (a) resolved target structure(s), for example, determined by crystallography or cryo-EM (Proschak et al., 2019).

### Superfolds

Similar folding of chain topologies of proteins with low sequence similarity; superfolds may form the basis for Supersites (Orengo et al., 1994; Russell et al., 1998).

### Superpatterns

Molecular-structural frameworks, entities, and/or elements that reoccur in Privileged ligands and form the molecular-structural basis of polypharmacology toward a set of structurally, functionally, and/or phylogenetically (un)related targets, particularly regarding the binding to Superfolds and Supersites (Stefan and Rafehi, 2023).

### Supersites

Supra-structural motifs within Superfolds that bind similar ligands/small-molecule drugs despite phylogenetic distance of the proteins considered; related to Chemoisosterism; supersites can be explored by Protein structure similarity clustering (Russell et al., 1998; Koch et al., 2004; Koch, 2011; Jalencas and Mestres, 2013b; Anighoro et al., 2014).

### Systems biology

Definition and mapping of entire biochemical regulation mechanisms, including monitoring the responses of Cellular networks and the Interactome to perturbations to understand complex interactions in biological systems (Azmi and Mohammad, 2014; McKie, 2016; Wang and Yang, 2022; Wu et al., 2022).

### Systems chemistry

Definition and mapping of networks of interacting small-molecular components that form new functions and obtain emergent properties at different hierarchical levels; systems chemistry emphasizes the boundary between prebiotic and biotic systems focusing autocatalytic systems, compartmentalized chemical systems, dynamic (or constitutional) Combinatorial chemistry, kinetic stability, self-assembly, self-maintaining, self-organization, self-replicating, self-reproducing (non-)metabolic networks, and thermodynamic equilibria (Altamura and Fiore, 2022).

### Systems pharmacology

*see* Network pharmacology.

### Target class repurposing

Similar to Target repurposing, but the actual target of interest is unknown, and it is only assumed to belong to a similar target class for which pharmacological tools exist; the term Target repurposing is often used in the pharmacological translation between species/organisms; related to Target hopping, target class repurposing is also used in the context of a transfer of knowledge from one protein family to another functionally and/or phylogenetically distant (known) protein family (Klug et al., 2016; Singh et al., 2020; Haupenthal et al., 2024).

### Target constellations

A group of functionally, inter-cellularly, and/or cell-type-linked proteins that elicit a physiological function (Wang and Yang, 2022).

### Target deconvolution

Retrospective Target identification after Phenotypic screening (High-content screening) (Terstappen et al., 2007; Bowes et al., 2012; Proschak et al., 2019).

### Target fishing, *in silico*

Computational prediction of compound–target interactions based on the chemical structure of a ligand/small-molecule drug using biologically annotated chemical databases; *in silico* target fishing is an integral part of Target identification at the very beginning of the drug development pipeline; several web tools exist to predict novel drug targets (Jenkins et al., 2006; Vulpetti et al., 2012; Wang and Xie, 2014; McKie, 2016; Ji et al., 2023).

### Target fishing, *in vitro*

Experimental setup using immobilized ligands/small-molecule drugs to identify novel binding partners by, for example, mass spectrometry, quantitative proteomics, or microarrays; *in vitro* target fishing is an integral part of Target identification at the beginning of the drug development pipeline; *in vitro* target fishing can be seen as reciprocal to Ligand fishing (Chen et al., 2020).

### Target hopping

The interaction between two or more targets through chemical space by sharing a similar set of ligands/small-molecule drugs without further interactions in physical or phylogenetic space and subsequent use of this set of ligands/small-molecule drugs to explore these drug targets (Paolini et al., 2006).

### Target identification

Discovery of a particular protein (family) or pathway with clear association to a pathological condition. Target identification represents the very beginning of the drug development pipeline.

### Target multiplicity

The organization of phylogenetically related protein superfamily members within a broad spectrum of evolutionary variation and functional diversity (McKie, 2016).

### Target phylogeny

Discipline which analyzes the likelihood of a ligand/small-molecule drug, which binds to one particular target protein, to also bind to phylogenetically related target proteins (Anighoro et al., 2014).

### Target profiling

*In silico* methodology to predict interactions between a panel of phylogenetically close and/or distant target proteins and (a) virtual ligand(s)/small-molecule drug(s); in the drug development pipeline, target profiling is used to discover unknown and/or unwanted Off-target effects; target profiling can be seen as reciprocal to Drug profiling (Jalencas and Mestres, 2013b; Antolin and Mestres, 2015).

### Target repurposing

Translation of knowledge (e.g., ligands) derived from a target (class) or group of targets to a known, functionally, and/or phylogenetically related target of interest; this term is often used in the pharmacological translation between species/organisms. Related to Target hopping and Target class repurposing (Paolini et al., 2006; Pollastri and Campbell, 2011; Klug et al., 2016; Singh et al., 2020).

### Target space

*see* Space.

### Target validation

Exploration and assessment of newly identified potential drug target(s) for critical aspects in drug discovery, such as assayability and druggability (Emmerich et al., 2021).

### Targeted polypharmacology

Anticipated development of ligands/small-molecule drugs that engage multiple targets with the aim of enhancing efficacy and safety (Morphy and Rankovic, 2006).

### Undruggability

The opposite of Druggability (Coleman and Rodon, 2021).

### Unselectivity

The opposite of Selectivity; also referred to as Non-selectivity or Cross-reactivity (Morphy et al., 2004; Morphy and Rankovic, 2005; Morphy and Rankovic, 2006; Ravikumar and Aittokallio, 2018).

### Unspecific effect

Biological effects that do not relate to the specific drug-target interaction but to other, unspecific interactions leading to the same experimental outcome/observation; also referred to as Non-specific effect.

### Virtual screening

Screening of diverse (Chemical space) or focused virtual compound libraries applying descriptors and/or fingerprints to obtain novel Chemotypes in the context of the anticipated targets; several virtual screening approaches have been developed to uncover polypharmacological ligands/small-molecule drugs, such as Computer-aided pattern analysis (C@PA) or Computer-aided pattern scoring (C@PS) (Peters, 2013; Namasivayam et al., 2021a; Namasivayam et al., 2021b; Namasivayam et al., 2022b, Stefan et al., 2024).

### Yet-to-be-drugged target

*see* Orphan target.

## 3 Concluding remarks

As sophisticated and mature the above terminology seems, there is no doubt that medicinal polypharmacology, with all its facets, is still at an early stage despite two decades of evolution (Anighoro et al., 2014; Proschak et al., 2019). Medicinal polypharmacology has attracted the attention of medicinal chemists, chemical biologists, clinical pharmacologists, and researchers from various other disciplines; however, the fact that it is still a small and young field limits its advancement.

Nevertheless, medicinal polypharmacology has an inherently multidisciplinary and translational character, bearing the unique chance to promote more integral and sophisticated strategies in modern drug development through international collaboration to 1) biologically assess, 2) structurally explore, and 3) clinically evaluate yet hidden drug targets and drug target combinations of the future.
